# Symptoms of Post-Traumatic Stress and Mental Health in a Sample of University Students: The Mediating Role of Resilience and Psychological Well-Being

**DOI:** 10.3390/ejihpe14080151

**Published:** 2024-08-05

**Authors:** Sílvia Ala, Francisco Ramos-Campos, Inês Carvalho Relva

**Affiliations:** 1 Department of Social Sciences, Life and Public Health Polytechnic Institute of Bragança, School of Health, 5300-121 Bragança, Portugal; 2 Research Group on Neuroscience and Psychiatric Illnesses in Instituto de Investigation Sanitaria Galicia Sur, 36213 Vigo, Spain; 3 Department of Personality, Assessment and Psychological Treatments, Faculty of Psychology, University of Salamanca, 37005 Salamanca, Spain; frc@usal.es; 4 Department of Education and Psychology, University of Trás-os-Montes e Alto Douro, 5000-801 Vila Real, Portugal; irelva@utad.pt; 5 Research Center in Sports Sciences, Health Sciences and Human Development (CIDESD), University of Trás-os-Montes e Alto Douro, 5000-801 Vila Real, Portugal; 6 Centre for Research and Intervention in Education (CIIE), University of Porto, 4200-135 Porto, Portugal

**Keywords:** pandemic, symptomatology mental health, anxiety, emotional exhaustion, post-traumatic stress symptomatology, psychological well-being, resilience, university students

## Abstract

The COVID-19 pandemic has been one of the most stressful events in recent times across the world. The long-term effect of these experiences raises several concerns, including the development of post-traumatic stress symptomatology. However, little is known about the psychological processes that mediate this association. The aim of this study was to explore the association of emotional exhaustion and anxiety in post-traumatic stress symptomatology, and the mediating role of resilience and psychological well-being in university students. A total of 526 university students of both sexes participated in this study, and they were aged between 17 and 62 years old. Symptoms of anxiety and emotional exhaustion were significantly higher in females, in contrast, males showed on average more resilience and psychological well-being. Additionally, participants with COVID-19 infection had higher levels of emotional exhaustion, anxiety, and PTSD. The results indicated that the variables were correlated with each other (*p* < 0.001). A conceptual model was confirmed that describes anxiety and emotional exhaustion as predictors, post-traumatic stress symptomatology as an outcome variable, and resilience and psychological well-being as mediators. Resilience and psychological well-being can be important protective factors for adaptive responses in stressful situations. The findings obtained in this study will provide a theoretical basis for designing targeted interventions to improve psychological health, whether for crisis intervention, the process of adapting to higher education, or for recovery plans from psychological trauma.

## 1. Introduction

The pandemic, which emerged at the end of 2019, caused by the Sar-Cov-19 virus, known as COVID-19, is characterized as a new, unique, multidimensional and potentially stressful factor with specific characteristics: global spread of unpredictable duration, individual effects in different areas of life, subjectively experienced loss of control, systemic effects on society and restrictions on health facilities and protective factors such as leisure activities or sports activities [1]. It has caused unprecedented disruption in the daily lives of people around the world [2], with adverse psychological and behavioral responses, both in the general population and in specific subgroups [1,3,4], namely university students [5,6].

However, even before the start of the pandemic, around 15% to 25% of university students worldwide suffered from one or more disorders during their academic training [7,8]. This is because the university setting is complex and some of its characteristics can maximize student suffering [9]. The demands and requirements of university life mean that students, from the moment they enter, must have complex cognitive and emotional resources to cope with the demands of this new environment. In addition, adolescence/young adulthood is considered a sensitive period for social development, autonomy and identity [10], and building a concept of self and psychological resilience, reinforcing that it is at this stage of development that human beings are most vulnerable to mental health problems [11,12]. This period involves inherent challenges with characteristics, as it is a time of transition and adaptation for students, characterized by situations of imbalance, discontinuity, anxiety and commitment to adaptation [13].

Thus, in a university context that is already characterized by academic, personal and social demands and requirements [14,15], the pandemic has made it even more complex, aggravating students’ vulnerability and triggering direct and negative effects on psychological well-being through negative emotional responses in the face of measures to contain the virus, such as lockdown, social distancing and the associated economic crisis [16]. In addition, students have had to deal with some emerging problems, such as adapting to a new context (online teaching), in which they have had to face issues of accessibility and connectivity, academic load, prolonged exposure to electronic devices, new demands on routines, organization of time and study strategies, fear of COVID-19 infection and precariousness in their professional future [14,17,18]—these are some aspects that can constitute significant stress factors, favoring the prevalence of emotional exhaustion, and anxiety and PTSD symptoms.

### 1.1. Psychological Impacts of the Pandemic: Emotional Exhaustion, Anxiety Symptoms and PTSD Symptoms

Emotional exhaustion is related to burnout syndrome, based on the theoretical model of Maslach et al. [19] and is one of the three dimensions of burnout (emotional exhaustion, depersonalization and dissatisfaction with performance) [20,21]. However, the most prevalent dimension among university students is emotional exhaustion, since the other two factors have not been observed in a significant or recurrent way [19,22,23].

Emotional exhaustion is characterized as a type of response to stressful situations that occur in different contexts, and is characterized by loss of energy, feelings of exhaustion and tiredness, and fatigue, resulting from intense emotional demands [24,25], such as role ambiguity and work overload. This leads to intense interactions and academic pressures [26], and culminates in feelings of incompetence [27,28] and an inability to fulfill academic responsibilities, thus affecting quality of life and psychological well-being [24].

The literature points to a significant prevalence of emotional exhaustion (manifested as emotional and cognitive fatigue) in university students, and it is associated with symptoms of anxiety and depression [22,23,29,30]. In addition, anxiety symptoms have been widely cited as the most common negative impact of the pandemic on university students [31,32], attributed to indicators of concern about personal health, the health of close friends and family, and the total disruption of personal, professional and mental health care routines [33,34,35]. In terms of analyzing the predictors, the results highlight online learning and the lack of moments of “isolation” to study, denoting the challenges experienced by students when it comes to finding moments with the necessary conditions to concentrate and study [33]. In the study by Fruehwirth et al. [35] using longitudinal data, the researchers reported that rates of moderate–severe anxiety increased by 39.8%, and rates of moderate–severe depression increased by 47.9% from before to the middle of the pandemic.

In this sense, anxiety is classified as a diffuse, unpleasant and vague feeling of apprehension, which is generated in anticipation of some event or danger, and is often accompanied by autonomous symptoms [36,37]. Anxiety disorders are commonly manifested by excessive fear and anxiety associated with behavioral disorders [28] and can be divided into trait anxiety (personality trait) and state anxiety, which indicates a physiological state that occurs when there is exposure to adverse situations, referring to an emotional state in the face of a temporary situation [29]. Thus, anxiety, as a manifestation of an emotion, is characterized by physical and psychological discomfort described by individuals as a feeling of intense and constant restlessness, nervousness and excessive worry [26], and can be linked to dysfunctional beliefs [27]. Anxiety is the anticipation of a future threat, with muscle tension, a state of vigilance (in preparation for future danger) and avoidance or apprehension behaviors. Thus, although anxiety symptoms are more severe and prominent in anxiety disorders, they are not exclusive to them and can occur in many other disorders [36]. It can therefore be the case that anxiety, as a symptom, is much more prevalent than anxiety disorders due to its cross-cutting nature, which cuts across psychiatric pathologies, medical pathologies and life events. Throughout this work, anxiety will be addressed as a symptomatology and not as a medical/psychiatric diagnosis (anxiety disorder).

In addition to emotional exhaustion and anxiety symptoms, university students showed a high incidence of PTSD symptoms during the pandemic, namely 35.5% [38,39]. In the study carried out by Williams et al. [40], the experience of isolation/confinement and loss of social interaction was compared by the participants as an experience of loss or “mourning”, encompassing feelings of loss of social/personal interaction, loss of routines, motivation, meaning and self-esteem. Stress and its impact on health are expected to increase the number of individuals with PTSD symptoms, as the symptoms may appear even more problematic and long-lasting [41], manifesting as the repeated experience of intrusion, avoidance and increased alertness, which are closely associated with PTSD symptoms.

PTSD, previously considered to be a type of anxiety disorder, is now seen by the Diagnostic and Statistical Manual of Mental Disorders (DSM-5), fifth edition [36], as part of trauma and stress-related disorders, which may lead to greater acceptance of PTSD as a disorder linked to external events and not as an anxiety-related mental illness. It is characterized by exposure to a concrete episode or threat of death, serious injury, or sexual violence (the individual may directly experience, witness, or know that it has happened to someone close to them [36]. The symptoms resulting from the pandemic can also be explained by adaptation disorder, which is a maladaptive response to a psychosocial stressor. The maladaptive response usually involves normal emotional and behavioral reactions that manifest more intensely than usual (considering contextual and cultural factors), causing marked distress, pre-occupation with the stressor and functional impairment [39,40]. Normally, this reaction can be temporary and manageable. However, when left unchecked, serious psychological imbalances can occur, and some people will develop PTSD and related symptoms [38].

### 1.2. The Mediating Effects of Resilience and Psychological Well-Being

Empirical evidence indicates that, in the face of adverse circumstances, university students have multifaceted ways of overcoming and coping, covering emotions, perceptions and experiences, which can mitigate mental health problems resulting from the COVID-19 outbreak. In this context, resilience and psychological well-being can act as protective factors [42,43], mitigating negative psychological impacts such as trauma, threats and high levels of stress [38,39,40].

Psychological resilience is characterized by the concentrated effort to achieve success in performing a task and the ability to overcome obstacles [44], tragedies, threats or other significant sources of stress [45], helping individuals to reduce vulnerability [46,47], preserve mental health, or recover after exposure to trauma in order to return to the pre-crisis state [48,49,50], especially during stressful events such as pandemics [51]. Levels of psychological resilience during the COVID-19 pandemic have been negatively associated with anxiety [52,53], less psychological trauma caused by stress [54], greater academic success [55], sense of psychological well-being [56], satisfaction with life, positive affect and emotional balance [57]. It may play a mediating role in the relationship between emotional exhaustion, anxiety symptoms and PSTP [27,58,59].

In addition to resilience, psychological well-being is also considered a protective factor, as individuals with this characteristic are more efficient at coping with stressful conditions [58] and less vulnerable to mental distress [60]. Positive mental health is defined as a state of well-being in which the individual realizes their own abilities, can cope with the normal stresses of life and can work productively and fruitfully [61], characterized by positive feelings and satisfaction with life [62]. Psychological well-being is, however, compromised when negative emotions are extreme or very long-lasting and interfere with the ability to be functional on a daily basis [63]. On the other hand, the presence of a state of psychological well-being can reflect a decrease in the symptoms of mental disorders [64].

Thus, as a stressful event, the pandemic has seriously affected the mental health of university students, but we cannot limit ourselves only to the clinical manifestations of the virus infection, as it is feared that its effects will manifest themselves in the short- and long-term [65], resulting in psychological trauma, going unnoticed or even untreated, leading to what Cardenas et al. [66] call a “parallel pandemic”, characterized by an increase in psychological suffering and symptoms. In particular, emotional exhaustion and anxiety symptoms [2] can have a negative impact on protective factors such as psychological resilience and psychological well-being, further aggravating psychological suffering [17,32,67,68] and the appearance of PTSD symptoms [69,70].

### 1.3. Sociodemographic Characteristics and Mental Health

With regard to prevalence and sociodemographic variables, some authors suggest that women experience more emotional exhaustion [17,22,29,30,71], anxiety symptoms [17,32,33,72,73,74] and PTSD [35,75,76,77] than men, and that women report lower levels of resilience and psychological well-being than men [75,78,79]. As for age, the level of mental health of older adults was higher than that of younger individuals [80], as was the level of psychological well-being [79,81,82]. The differences found in younger people with more reported symptoms may be because their cognitive schemas about themselves and the world are not yet consolidated and, as such, they are more susceptible to disruption and change [10]. The reduction and/or loss of income caused by the measures to mitigate the pandemic also seem to be related to possible implications in the exacerbation of stress and anxiety symptoms [83], in the reduction of the quality of family life, and consequently in the well-being of students [71,78,79].

Contact with nature, such as by living in rural areas or frequenting urban green spaces (e.g., city parks or public or private gardens) and other natural spaces (e.g., beaches or riverside areas) provide opportunities for relaxation and have a salutogenic effect on mental health, such as by reducing psychophysiological responses to the stress caused by confinement [83,84].

Therefore, in addition to the individual and contextual factors involved in the mental health process, some specific aspects of university life and the limitations imposed by the pandemic may constitute risk or protective factors for the mental health of university students. 

### 1.4. Objectives and Hypotheses

The aim of this study is, firstly, to identify the association of sociodemographic variables (sex, family income, COVID-19 testing and area of residence during contingency measures) on the relationships of the theoretical model presented. The study aims, secondly, to analyze the association of emotional exhaustion and anxiety symptoms, resilience and psychological well-being on PTSD symptoms. Thirdly, it aims to identify the mediation role of resilience and psychological well-being between emotional exhaustion and anxiety symptoms on PTSD symptoms.

In pursuing our objectives, we intend to contribute to the literature by exploring the dimensions that can mitigate the impact and changes in mental health in university students, whether due to the effects of the transition/adaptation to higher education or in future similar situations, thereby enhancing the continuation of new discoveries to design interventions with measures that promote well-being and adjustment at all levels, and contributing to the transformation of universities as places that promote health and psychological well-being.

Based on these theoretical premises and previous research, we formulated the following hypotheses:

**H1:**  *Sociodemographic factors are associated with greater emotional exhaustion, symptoms of anxiety and PTSD. Specifically, we expect to find higher levels of emotional exhaustion, anxious symptomatology and PTSD symptomatology in (i) females, (ii) participants with decreased family income, (iii) participants who tested positive for COVID-19 and (iv) students who have experienced contingency measures in urban areas.*

**H2:**  *The variables of sex, COVID-19 test, environment/place of residence during contingency, emotional exhaustion, anxiety symptomatology, resilience and psychological well-being are associated with PTSD symptoms.*

**H3:**  *Resilience and psychological well-being are negatively associated with PTSD symptoms.*

**Figure 1:**
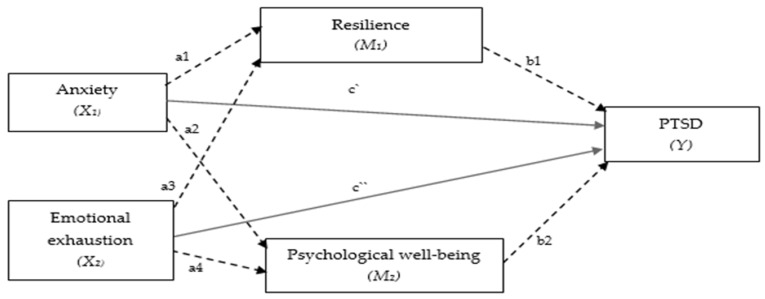
Hypothetical conceptual model for the mediating roles of resilience and psychological well-being in the relationship between anxiety symptomatology and emotional exhaustion in PTSD symptomatology.

**H4:**  *Resilience and psychological well-being function as mediators in the relationship between emotional exhaustion and anxiety symptomatology in PTSD symptomatology. The hypothetical model (Figure 1) aims to explore whether and how the association between emotional exhaustion and anxiety symptoms change by introducing resilience and psychological well-being as mediators. In our hypothetical mediation model (Figure 1), the total effects included two paths of direct effects of X1 (anxiety symptomatology) and X2 (emotional exhaustion) on Y (PTSD symptomatology) (path c′ and c″), and four indirect paths of X1 and X2 mediated through M1 (resilience) and M2 (psychological well-being) (path a1b1 + path a3b1 + path a2b2 + path a4b2).*

## 2. Materials and Methods

### 2.1. Sample

The sample consisted of 526 university students, 19% male and 81% female, aged between 17 and 62 (*M* = 21.42; *SD* = 5.79). The most representative areas of study were health sciences (31%) and social and behavioral sciences (41%). In terms of academic progress, 42.6% of the students were first-year undergraduates, 18.6% were second-year undergraduates and 15.8% were third-year undergraduates. Regarding the question about monthly household income during the pandemic, 56.3% remained the same, while 38.2% of the participants’ income decreased. In addition, 69.8% tested positive for COVID-19 and 67.9% of participants perceived changes in mental health during the measures imposed by COVID-19. Finally, 50.4% of the subjects were quarantined in rural areas, and 49.6% were in urban areas.

### 2.2. Instruments

A questionnaire was drawn up with sociodemographic data and the scales under study. A semi-structured questionnaire on sociodemographic profile and experience with contingency measures included the participant’s sex, age, year and area of study, place of residence during the contingency measures (rural/urban), household income during the contingency measures (decreased, maintained, or increased) and COVID-19 infection test (positive or negative).

The instruments used had no direct link to the pandemic. However, it was added to the survey instruments that respondents should focus on the restrictions/changes imposed by the COVID-19 Pandemic and (for example in PCL-5) to each item specified as a stressful event in relation to the COVID-19 pandemic. An example of an item is: “Repeated and disturbing memories, thoughts or images of a stressful experience of the COVID-19 lockdown”.

To assess post-traumatic stress disorder symptomatology, the Post-traumatic Stress Disorder Checklist for DSM-5 (PCL-5) ([85]-Portuguese version by [86]) was used. This is a multidimensional self-report instrument, made up of 20 items which aim to assess the severity of the 20 PTSD symptoms present in the last month and/or dichotomously measure the presence of a diagnostic condition, according to the DSM-5 criteria. The items are answered on a five-point Likert scale, ranging from 0 (none) to 5 (extremely) [85,87]. These are divided into four different symptom groups: intrusions, avoidance, negative changes in cognition and mood, and significant changes in activation and reactivity [36]. The PCL-5 was adapted and translated for the Portuguese population by Carvalho et al. [86] and has good psychometric properties, with high internal consistency (α = 0.94). The instrument refers to a specific traumatic event and the administration format used was to ask participants to identify the COVID-19 pandemic as a defining event. Our confirmatory factor analysis indicated that the fit of the data to the proposed theoretical model led to the following values: SRMR = 0.07, CFI = 0.89, RMSEA = 0.09, χ^2^_(167)_ = 932.046, *p* < 0.001, χ^2^/df = 5.58 and alpha = 0.95.

Resilience was assessed using the Portuguese version of the Connor–Davison Resilience Scale-10 (CD-RISC-10, [88,89]). This scale consists of 10 items that assess on a five-point Likert scale (0 = “Not true”; 4 = “Almost always true”) how much each of the items applied to the respondents throughout the previous month. The total score on the scale ranges from 0 to 40, with higher scores indicating greater resilience. A score below 29 (quartile 25 associated with the North American population) is an indicator of low resilience. The original version of the CD-RISC-10 scale [60] had good psychometric properties (α = 0.89) in the validation study for the Portuguese population [61] (α = 0.85). The confirmatory factor analysis indicated that the data fit the proposed theoretical model with the following values: SRMR = 0.034, CFI = 0.93, RMSEA = 0.075, χ^2^_(35)_ = 138.874, *p* < 0.001, χ^2^/df = 3.97 and alpha = 0.84.

The reduced version of the Mental Health Inventory (MHI-5), validated for the Portuguese population [90,91], was used. The MHI-5 is part of the National Health Survey and is a mental health instrument recommended by the WHO for use in population health studies. It consists of five items on mental health, and the results are classified using an indicator that measures the probable existence of psychological distress. It is a self-administered questionnaire that aims to assess mental health from a perspective that includes a positive dimension (psychological well-being, positive mental health status) and a negative dimension of mental health (psychological distress, psychological suffering). In this study, only the positive dimension of positive psychological well-being was used as a mediator. The MHI-5 expresses the same results as the long version, with good psychometric properties (α = 0.80). Confirmatory factor analysis indicated that the data fit the proposed theoretical model with the following values: SRMR = 0.07, CFI = 0.95, RMSEA = 0.14, χ^2^_(4)_ = 49.811, *p* < 0.001, χ^2^/df = 12.45, alpha = 0.81 (total scale) and the psychological well-being subscale obtained an alpha = 0.73.

To measure emotional exhaustion, the Emotional Exhaustion Scale [29], validated for the Portuguese population [72], was used, and it consisted of 10 items that measured emotional exhaustion. It scored on a Likert-type scale ranging from 1 (rarely) to 5 (always), when considering the last 12 months of classes. The ECE is a unidimensional scale with an acceptable level of internal consistency, with an alpha coefficient of 0.89 [72]. The confirmatory factor analysis indicated that the fit of the data to the proposed theoretical model was as follows: SRMR = 0.07, CFI = 0.93, RMSEA = 0.10, χ^2^_(31)_ = 206.637, *p* < 0.000, χ^2^/df = 6.66 and alpha = 0.89.

Finally, the General Anxiety Disorder (GAD-7, [92], validated for the Portuguese population [93]), was used to assess anxiety. The answers were given on a scale ranging from 0 to 3, referring to the frequency of symptoms. The total scores ranged from 0 to 21, with higher scores indicating a greater severity of anxiety symptoms; the original scale had good psychometric qualities (α = 0.91). Confirmatory factor analysis indicated that the data fit the proposed theoretical model with the following values: SRMR = 0.017, CFI = 0.99, RMSEA = 0.05, χ^2^_(12)_ = 28.157, *p* < 0.005, χ^2^/df = 2.35 and alpha = 0.88.

### 2.3. Procedures

This study was carried out in accordance with the latest version of the Declaration of Helsinki, the European Union’s General Data Protection Regulation and the Code of Ethics and Deontology for Research of the Portuguese Psychologists’ Association. Authorization was obtained from the Ethics and Data Protection Committee of the Polytechnic Institute of Bragança (Opinion no. 134/2023-453683). 

The sample was collected in the north of Portugal and the data were collected during the pandemic. In the 2022/2023 school year at the end of the 1st semester, we no longer had restrictions imposed related to the pandemic, however, in some questions the participants were asked to focus on their experiences of the restrictions and changes imposed by the COVID-19 pandemic. The survey was conducted online using the “Limesurvey” platform. Informed consent was obtained during the online survey, the average time to complete the survey was 15 min.

### 2.4. Statistical Analysis

This is a cross-sectional study in terms of the data collection method. Demographic and psychological variables are presented as descriptive statistics. The sample size was tested in G*Power 3.1.9.7, using an a priori power analysis with effect size d = 0.5, significance level of 0.05, power of 0.95, eight predictors and a minimum of 54 participants. 

Data processing involved building the database and observing the normality values of the distribution (asymmetry and kurtosis) [94]. All questionnaires were validated and showed no missing values. In addition, we calculated the Mahalanobis distance (*p* < 0.001) for all scores to detect and omit multivariate outliers. The statistical program Statistical Package for Social Sciences-SPSS, version 28 (IBM Corp., Chicago, IL, USA, released in 2021), was used.

We carried out 1st-order confirmatory factor analyses (CFAs) for the results derived from the use of the instruments. The variables under study were characterized using descriptive statistics and frequencies. The data were expressed as mean (M) ± standard deviation (SD). The *t*-test for independent samples was used to analyze differences between two groups, and one-way ANOVA was used to explore differences between three or more groups. Hierarchical regression analysis was used to identify the predictors of PTSD symptomatology scale scores, with a *p*-value of less than 0.01 being considered significant. Pearson’s correlation was used as a preliminary path analysis to determine the relationships between the selected variables. According to Cohen [95], the correlation is weak when *r* = 0.10 to 0.29 or *r* = −0.10 to −0.29, moderate when *r* = 0.30 to 0.49 or *r* = −0.30 to −0.49, and strong when *r* = 0.50 to 1.0 or *r* = −0.50 to −1.0.

Hierarchical regression analysis was used to evaluate the relationship between each of the variables (sex, COVID-19 test, place of residence during contingency measures, anxiety symptoms, emotional exhaustion, resilience and psychological well-being) using the *R*^2^ value and the standardized β coefficient. The objective was to calculate the impact of variables on post-traumatic stress symptoms. The variables of sex, COVID-19 test and place of residence during contingency measures were recorded in dummy variables.

The hypothetical model was tested to analyze the mediating effect of resilience and psychological well-being between simple paths in the multiple mediation model (anxiety symptomatology and emotional exhaustion were presented as predictors, and PTSD symptomatology as the dependent variable). Several steps were developed in the mediation test, namely the role of direct and indirect effects. The path analysis model was run using the Statistical Package for Social Sciences-SPSS AMOS, version 26 [96]. We conducted a mediation model using bootstrapping analyses and a 90% bias-corrected confidence interval with a resampling procedure of 5.000 bootstrap samples. The mediation effect test is significant if it does not contain zero below the 90% confidence interval (CI).

The model was evaluated using the chi-square test, (χ^2^), and a comparative fit index (CFI), normed fit index (NFI), Tucker–Lewis index (TLI) and root mean square error of approximation (RMSEA) indices were used to examine the quality of model fit. The reference values for acceptable fit were CFI ≥ 0.90 and RMSEA < 0.10 [94]. All results were analyzed based on a significance value of *p* < 0.05.

## 3. Results

### 3.1. Analyses–Descriptives, t-Test, One-Way ANOVA

Descriptive statistics related to COVID-19 post-traumatic stress disorder, resilience, psychological well-being, anxiety and emotional exhaustion are presented in Table 1. Overall, it should be noted that the participants obtained moderate scores in the dimensions of psychological well-being and anxiety, and high scores in post-traumatic stress symptomatology, resilience and emotional exhaustion. All the measures met the assumptions of normality. Regarding reliability, all the scales showed moderate-to-high reliability values, ranging from 0.73 (psychological well-being subscale) to 0.95 (PTSD).

Table 2 shows differential analysis regarding gender and whether participants tested positive or negative for COVID-19 using the independent samples *t*-test (*p* < 0.05). At the level of emotional exhaustion, the results indicate that there was a statistically significant difference between the mean test scores of females (*M* = 30.83; *SD* = 8.00) and males (*M* = 27.06; *SD* = 8.46); the test revealed a *t*-statistic = −4.19, *p* = <0.001; *d* = −0.47, with a medium effect size. There were also significant differences in terms of anxiety symptoms (*t* = 2.201, *p* = 0.03; *d* = −0.245) and females (*M* = 8.99, *SD* = 4.77) had a higher test score than males (*M* = 7.81, *SD* = 5.07). Symptoms of anxiety, emotional exhaustion and symptoms of PTSD were, on average, higher in participants who tested positive for COVID-19 than in participants who tested negative, however the values were not statistically significant.

The independent *t*-test did not show statistically significant differences between the means of students who lived in rural areas compared to those who lived in urban areas during contingency measures imposed by the pandemic.

A differential analysis of family income during contingency measures (decreased, maintained, or increased) was carried out using one-way analysis of variance (ANOVA) of independent samples. At the level of emotional exhaustion, the results indicate that there are no statistically significant differences between the mean scores of the family income groups [(F_(522, 3)_ = 1.937; *p* = 0.123)], and the same was observed for anxiety symptoms (*p* = 0.404), resilience (*p* = 0.362), psychological well-being (*p* = 0.109) and symptoms of PTSD (*p* = 0.081).

### 3.2. Correlations between the Study Variables

The association between emotional exhaustion and anxiety was found to be a strong, positive and significant correlation (*r* = 0.500, *p* < 0.01). Resilience was moderately negatively correlated with symptomatology anxiety (*r* = −0.346, *p* < 0.01) and emotional exhaustion (*r* = −0.360, *p* < 0.01). In the association between psychological well-being, there was a strong and significant negative correlation with anxiety (*r* = −0.528, *p* < 0.01) and a moderate one with emotional exhaustion (*r* = −0.339, *p* < 0.01). The correlation with resilience was positive and weak (*r* = 0.270, *p* < 0.01). Finally, post-traumatic stress symptomatology correlated strongly and positively with anxiety symptomatology (*r* = 0.649, *p* < 0.01) and moderately with emotional exhaustion (*r* = 0.481, *p* < 0.01), and correlated negatively and moderately with resilience (*r* = −0.347, *p* < 0.01) and psychological well-being (*r* = −0.437, *p* < 0.01). The results of the correlations and the respective means and standard deviations are shown in Table 3.

### 3.3. Hierarchical Regression Analyses

The hierarchical regression analysis was conducted to explore the impact of demographics (gender, place of residence during contingency measures, COVID-19 tests), emotional exhaustion, anxiety symptomatology, emotional resilience and well-being are psychological factors on PTSD symptomatology, in which the independent variables are inserted into blocks, where each independent variable is evaluated in terms of what it adds to the prediction of the dependent variable [97] (Table 4). 

Preliminary analyses were performed to ensure that the assumptions of normality, linearity, multicollinearity and homoscedasticity were not violated. We proceeded with a hierarchical approach in five blocks.

The model predicted PTSD symptomatology by 47% (*R* = 0.47, Δ*R*^2^ = 0.47, F_(7,518)_ = 66.71, *p* < 0.001). The independent variables with significant values, emotional exhaustion (*β* = 0.352, *p* < 0.001), anxiety symptoms (*β* = 1.650, *p* < 0.001); resilience (*β* = −0.263, *p* = 0.005) and psychological well-being (*β* = −0.869, *p* < 0.001), remained as predictors in the final model. The negative value on the resilience and psychological well-being factors indicates that when individuals have higher resilience and psychological well-being levels, they are less likely to have PTSD symptoms.

### 3.4. Association of Anxiety and Emotional Exhaustion with PTSD: The Mediating Role of Resilience and Psychological Well-Being

Path analysis models were used to analyze the effect of anxiety and emotional exhaustion as predictors, resilience and psychological well-being as mediators, and PTSD as a target variable on the sample (*n* = 526).

**Figure 2:**
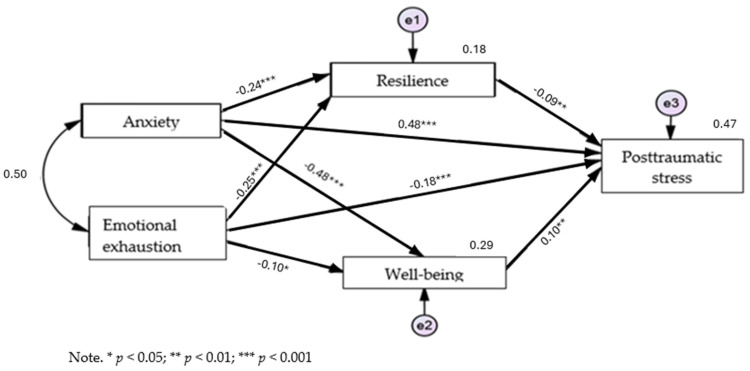
Representative model of the mediating role of resilience and psychological well-being in the association between anxiety, emotional exhaustion and PTSD symptomatology.

The results of the path analysis examining the mediating role of resilience and psychological well-being in the associations between anxiety, emotional exhaustion and PTSD indicated a good fit for the data (χ^2^_(1)_ = 3.963, *p* = 0.046, χ^2^/df = 3.963, CFI = 0.996 GFI = 0.997 RMR = 0.265, RMSEA = 0.075). Figure 2 shows the standardized path coefficients for the model tested. 

Regarding the initial model referring to anxiety and emotional exhaustion in the prediction of PTSD, it was observed that anxiety positively predicts PTSD (*β* = 0.55), and emotional exhaustion positively predicts PTSD (*β* = 0.21). The previous correlations were maintained after introducing the mediating variables of resilience and positive well-being, and there was a decrease in the initial direct effect of anxiety (*β*_initial_ = 0.55; *β*_end_ = 0.48) and emotional exhaustion (*β*_initial_ = 0.21; *β*_end_ = 0.18) (see Table 4) on PTSD.

Thus, the results of the model were subsequently used to test the study’s hypotheses. As presented in Figure 2, the total effect including a direct effect pathway (pathway c′) of anxiety (*β* = 0.48, *p* < 0.001; [0.405; 0.545]) and the total effect including a direct effect pathway (pathway c″) of emotional exhaustion (*β* = 0.18, *p* < 0.001; [0.107; 0.244]) were significant predictors of the PTSD factor. However, anxiety symptoms had a stronger effect than emotional exhaustion.

Anxiety symptoms had a negative, significant and stronger effect on psychological well-being (*β* = −0.48, *p* < 0.001, [−0.536; −0.418]). Emotional exhaustion, on the other hand, had a negative, significant and stronger effect on resilience (*β* = −0.248, *p* < 0.001, [−0.321; −0.025]).

It was observed that anxiety symptoms and emotional exhaustion exert a negative and significant indirect effect on PTSD symptoms through the total mediation of resilience (path a1b1 + path a3b1) (*β* = −0.09, *p* < 0.01; [−0.139; −0.034]) and psychological well-being (path a2b2 + path a4b2) (*β* = −0.10, *p* < 0.01; [−0.156; −0.038]). 

The hypotheses (H3 and H4) were confirmed through mediation analysis. This means that the relationship between anxiety symptomatology and emotional exhaustion as comorbidities resulting from COVID-19 stress and PTSD symptomatology results was fully mediated by resilience and psychological well-being. The indices associated with the direct and indirect effects resulting from the model in Figure 2 are shown in Table 5.

## 4. Discussion

The objectives of this study were to analyze the impact of emotional exhaustion, anxiety symptomatology, resilience and psychological well-being on the symptomatology of PTSD, and to identify the mediation of resilience and psychological well-being on the symptomatology of PTSD in university students. The study was conducted in the first semester of the 2022/2023 school year, and in this period in Portugal we no longer had containment measures (quarantine and online education) imposed for the COVID-19 pandemic.

In the analysis of our sample, university students presented higher mean values for emotional exhaustion, psychological resilience and PTSD symptoms. Females showed higher levels of anxiety symptoms and emotional exhaustion, and these results are in line with other studies [17,24,59,71,74,75,77,98,99]. In contrast, males showed higher levels of resilience and psychological well-being, and other studies have shown similar patterns [82,100]. These differences regarding sex may be because this variable cannot be limited to a reductive understanding as a biological variable, but rather encompasses a combination of biological, psychosocial and social factors. Some differentiating characteristics can be influenced by sociocultural construction that takes place through a process of socialization, in which one is influenced by gender norms, rules, stereotypes and expectations that can vary at a cultural level and over time [101]. Socialization can affect mental health in terms of diagnosis, prognosis, treatment and comorbidity [102]. From a psychosocial perspective, how women experience certain feelings, such as anxiety, stress and fear is normalized and they are expected to express them. As for men, the reinforcement of the suppression of certain emotions and the social expectation that they should be strong and courageous can lead to denial, making it difficult to externalize and consequently difficult to seek help. Another explanatory factor is that exposure to stress during puberty may have stronger effects on females, including a higher risk of developing mood- and stress-related psychopathologies such as depression, anxiety and post-traumatic stress symptomatology [103]. Changes in physiological factors, such as the brain structures responsible for emotional regulation [104], cortisol, genetic vulnerability [74] and hormonal changes can increase vulnerability to stress at an emotional level, while stress in men has an impact at a cognitive level [102]. The political, economic and social situation can also have a differential effect on the mental health of women and men [102]. These differences emphasize the importance of considering the biopsychosocial model of health and the perspective of gender when conceptualizing, researching and intervening in mental health.

H1 was partially supported because, among the sociodemographic variables, only females presented statistically significant and higher values of emotional exhaustion [17,22,29,30,71] and symptoms of anxiety [17,32,33,72,73,74]. Symptoms of anxiety, emotional exhaustion and PTSD symptoms were, on average, higher in participants who tested positive for COVID-19 than in participants who tested negative. Although the differences were not statistically significant, students who lived in rural areas during quarantine measures, presented higher symptoms of anxiety and PTSD, and lower resilience than students living in urban areas, perhaps due to more precarious economic conditions and fewer resources for access to health services [105]. Another reason may be because urban areas are more densely populated, which generated more concerns during the outbreak of COVID-19; equally, public health entities and policy makers may have invested more in informational messages about COVID-19 in big cities. As a result, rural areas may have been neglected. This may be the reason why university students living in small towns and villages reported worse mental health outcomes than those living in large cities. In the future, these factors should be considered to provide health support to university students living in rural areas [106]. However, students living in rural areas had less emotional exhaustion and better psychological well-being than colleagues living in urban areas. Our results may be due to the fact that, in Portugal, during contingency measures, residents were allowed to go out for short periods to walk pets and exercise outdoors (for example, walking), making it possible to be in contact with nature [107]. These results suggest that the use of nature, particularly in urban areas, can be used to improve mental health [83,84].

In the correlations of the variables under study, PTSD symptoms, emotional exhaustion and anxiety symptoms were positively related. On the other hand, the variables negatively related to anxiety symptoms were resilience and psychological well-being. The results were statistically significant, but it was the anxiety symptomatology that showed a stronger correlation with the symptoms of PTSD. These results go against the literature in the sense that the most common symptom of all anxiety disorders is the experience of anxiety, which may be cognitive or physical, and which is expressed in the symptomatology of PTSD as conditioned responses of anxiety, for example as the avoidance of situations that are reminders of the traumatic situation/event which, in turn, is negatively reinforced in order to avoid anxiety [36], all the while exacerbating the symptomatology of PTSD.

Hypothesis H3 was confirmed—resilience and psychological well-being were negatively associated with PTSD symptomatology [55,108,109,110,111,112,113], emphasizing the role of resilience and psychological well-being as protective factors, and individuals with these characteristics are more efficient in coping with stressful conditions [58] and less vulnerable to mental suffering [60,112]. 

In the analysis of the impact of sociodemographic data (sex, place of residence during contingency and COVID-19 test), emotional exhaustion, anxiety symptoms, emotional resilience and psychological well-being on the symptomatology of PTSD, we tested five models, the final one of which pre-determined significantly the symptoms of PTSD. The explained variance of the model was 47%, which is to say the symptoms of PTSD can be explained by the predictive variables (emotional exhaustion, symptoms of anxiety, resilience and psychological well-being).

Four hypotheses were tested, and a conceptual model was developed that described emotional exhaustion and anxiety symptomatology as predictors, PTSD symptomatology as an outcome variable and resilience and psychological well-being as mediators. The hypotheses were confirmed by multiple linear regression and SEM analysis.

After introducing the mediating variables of resilience and positive well-being, there was a decrease in the initial direct effect of anxiety symptomatology (*β*_initial_ = 0.55; *β*_end_ = 0.48) and emotional exhaustion (*β*_initial_ = 0.21; *β*_end_ = 0.18) on PTSD symptomatology. Furthermore, the direct effect controlled by the (standardized) mediators was significant.

As expected, resilience symptomatology and psychological well-being were found to have negative mediating effects [55,77,98,104], confirming the mediating role in the relationship between emotional exhaustion, symptomatology anxiety and PSTP [60,114]. The results highlight the importance of creating favorable learning environments for promoting students’ resilience and well-being and reducing their vulnerability [107]. Resilient persons can identify positive meanings in adversity [58], reflecting the ability to solve problems, overcome adversity with positive emotions and promote psychological health [59]. On the other hand, a decrease in resources, both in terms of psychological well-being and personal, social and contextual coping strategies is associated with increased levels of PTSD.

COVID-19 had severe effects on mental health and was responsible for a new type of traumatic stress, featuring multiple stressors, and which is continuous, involving changes in routine, contingency measures (isolation, online classes and confinement), fears of present or future infection, economic stressors and stressors related to confinement, and mourning for those lost [83]. Regarding the averages of resilience, psychological well-being and PTSD, the results were similar results to those of other studies [51,71].

### Limitations, Practical Implications and Suggestions for Future Studies

Some limitations can be highlighted. First, the use of a cross-sectional design limits the conclusions that can be drawn about causal relationships, since we do not have data from the sample before the COVID-19 outbreak. As our results are exploratory, it is recommended that future research opts for a larger, longitudinal sample to better explain the associations between anxiety and emotional exhaustion in PTSD, and the mediating roles of resilience and psychological well-being. Our sample lacks heterogeneity at the sex level (81% female) and the sample consisted of university students only from northern Portugal, limiting the generalization of the results, thus it is not representative of Portuguese university students.

Given the duration and extent of the pandemic, the pandemic could be associated with a complex and continuous trauma, not resulting in a single diagnosis but rather a comorbidity syndrome profile, describing the complex picture of the cumulative impact of different types of continuous and prolonged COVID-19 stress. Although the concept of PTSD is complex and has recently been codified in the ICD-11 [37], it may not be sufficient to describe the true mental health impact of the cumulative stressors of COVID-19 [51]. Therefore, the COVID-19 pandemic as a traumatic stress experience requires a paradigm shift in traumatic stress research, showing the limitations of current diagnoses, not only to understand the pandemic as a traumatic event, but also because the pandemic forces us to recognize a possibly new type of mass trauma. This requires a new perspective on the type/diagnosis of trauma and its implications [115]. This study therefore complements existing work by focusing on COVID-19 as an explicit stressor in a complex environment, such as the pandemic in the academic context. These results are also important in situations that make the switch to digital learning necessary and cause stress among students (e.g., wars and environmental disasters). 

Regarding recommendations and practical implications, this study has reinforced the need for the work of clinical and educational psychology to be geared towards promoting psychological well-being among university students and all those in education. Taking as guidelines the 2030 Agenda [116,117], mental health affects all areas of life and is interconnected with various aspects of the Sustainable Development Goals (SDGs). Reinforcing this is the analysis of SDG #3 (Health and Well-being), which recognizes the importance of promoting mental well-being as an integral part of general health. More specifically, target 3.4 states that, by 2030, we should have reduced premature mortality from non-communicable diseases by one third through prevention, treatment and the promotion of mental health and well-being. In addition, SDG #4 (Quality Education) highlights the need to promote safe and healthy school environments, including adequate psychosocial support for students. However, the promotion of mental health requires joint efforts and is key to developing effective strategies, sharing resources and experiences, and ensuring the successful implementation of policies and programs aimed at mental health [118].

It is undeniably important for universities and polytechnic institutes to have a psychological support office in their social action services, focused on promoting adaptation and academic success, considering the three moments of ecological transition that young people go through during higher education: entry (adaptation), attendance and exit (transition to the world of work) [119].

Thus, the scientific literature identifies initiatives and proposals for pedagogical, emotional and instrumental support for students, mainly: (a) Transition support plans: support management and stress-control programs, the development of study methods, the development of personal, social and academic skills [120], a focus on investment in life skills training: composed of ten skills (self-awareness, communication skills, interpersonal relationships, decision making, problem solving, creative thinking, critical thinking, empathy and emotional management [121]; (b) psychology consultations: therapeutic groups focused on specific symptoms, workshops and the acquisition of socio-emotional skills [122] in these therapeutic groups where the dynamics must assume playful, creative, and sensory interactions and relaxation methodologies [123]; finally (c) deliver educational and training programs for gatekeepers to all those involved in education so that non-teachers, teachers and students recognize warning signs of emotional crises and psychosocial risks [124], and promote programs and extracurricular activities that promote self-care and health literacy [125].

In the future, longitudinal studies should adopt large samples and a greater gender balance to explore the impact of variables on PTSD symptomatology. Additionally, it will be important to explore the perception that environmental variables such as social support can have on PTSD symptomatology when using a qualitative approach. 

## 5. Conclusions

This study intended to contribute findings to the existing literature that has focused on the relationship between PTSD symptomatology, anxiety symptomatology and emotional exhaustion as the results of exposure to a stressful event (the COVID-19 pandemic). To our knowledge, no other study had explored the symptoms of anxiety and emotional exhaustion as comorbid for the development of PTSD symptoms, and with the mediating role of resilience and psychological well-being. As expected, resilience and psychological well-being act as mediators between anxiety symptomatology, emotional exhaustion and PTSD symptomatology. Thus, this study contributed scientific insights to our understanding of the impact of stressful experiences on the mental health of university students. Our cross-sectional study suffered from the limitation of not being able to establish causal relationships. Future studies could explore causal relationships through longitudinal or experimental study designs. However, more studies should be conducted to deepen this theme, which is extremely important for the prevention and mitigation of the impact of changes to the mental health of university students due to the transition/adaptation to higher education or to future pandemic situations. In addition, this study implies the importance of contact with nature as a tool for promoting physical and mental health, especially in crisis situations. To promote the mental health of university students, academic stress should be addressed. It is also important to design interconnections with measures to promote well-being and adjustment at all levels, contributing to the transformation of universities as places that promote health and psychological well-being.

## Figures and Tables

**Table 1:** Descriptive statistics for research variables (*n* = 526).

Variables	*M*	*SD*	Min	Max	Range	Skewness	Kurtosis	Reliability
Symptomatology of PTSD	22.02	16.667	0	76	76	0.669	−0.243	0.95
Resilience	24.12	5.707	8	40	32	0.267	−0.062	0.84
Psychological well-being	6.75	1.909	2	12	10	0.387	−0.356	0.73
Symptomatology anxiety	8.79	4.868	0	21	21	0.600	−0.242	0.88
Emotional exhaustion	30.12	8.210	10	50	40	−0.122	−0.334	0.89

**Table 2:** The measured dimensions of the study were the differences between the sample of men vs. women, the positive COVID-19 test vs. negative COVID-19 test, and if participants resided in rural areas during contingency measures vs. residing in urban areas.

Dimension		Sex					Test COVID-19					Place of Residence during Contingency Measures				
		M	F	*t*	*p*	Cohen’s *d*	Yes	No	*t*	*p*	Cohen’s *d*	Urban	Rural	*t*	*p*	Cohen’s *d*
Symptomatology of PTSD	*M*	19.58	22.56	−1.61	0.11	−0.18	22.78	20.18	1.78	0.08	0.17	21.78	22.25	−0.324	0.75	16.68
	*SD*	16.55	16.67				17.52	14.44				16.68	16.68			
Resilience	*M*	24.92	23.90	1.63	0.10	0.18	23.84	24.66	−1.52	0.13	−0.14	24.58	23.67	1.842	0.07	5.69
	*SD*	5.59	5.68				5.66	5.68				5.63	5.75			
Psychological well-being	*M*	6.81	6.73	0.36	0.72	0.04	6.74	6.77	−0.20	0.84	−0.02	6.80	6.69	0.661	0.51	1.91
	*SD*	1.78	1.94				1.89	1.97				1.94	1.88			
Symptomatology anxiety	*M*	7.81	8.99	−2.20	**0.03**	−0.24	8.76	4.98	−0.02	0.98	−0.002	8.41	9.17	−1.794	0.07	4.85
	*SD*	5.07	4.77				8.77	4.52				4.85	4.86			
Emotional exhaustion	*M*	27.06	30.83	−4.19	**<0.001**	−0.47	30.31	8.35	0.85	0.39	0.08	30.58	29.67	1.273	0.20	8.20
	*SD*	8.46	8.00				29.65	7.89				8.09	8.30			

Note. Significant coefficients are in bold (*p* < 0.05; *p* < 0.01).

**Table 3:** Correlations between variables, means and standard deviations.

Variables (*n* = 526)	1	2	3	4	5
1. Symptomatology anxiety	-				
2. Emotional exhaustion	0.500 **	-			
3. Resilience	−0.346 **	−0.360 **	-		
4. Psychological well-being	−0.528 **	−0.339 **	0.270 **	-	
5. Symptomatology of PTSD	0.649 **	0.481 **	−0.347 **	−0.437 **	-
*M* ± DP	8.79 ± 16.66	30.12 ± 8.21	24.12 ± 5.70	6.75 ± 1.90	22.02 ± 16.66

Note. ** *p* < 0.01. Correlation is significant (2-tailed).

**Table 4:** Predictive role of demography, emotional exhaustion, symptoms of anxiety, resilience and well-being on the symptomatology of PTSD.

*Symptoms of PTSD*								
	Variables	*R* ^2^	Δ*R*^2^	*β*	*SE*	*Beta*	*t*	*p*
Block 1	Sex	0.010	0.004	−0.710	1.380	−0.017	−0.514	0.607
	Place of residence during contingency measures			−0.697	1.077	−0.021	−0.647	0.518
	COVID-19 testing			−2.114	1.160	−0.058	−1.823	0.069
Block 2	Emotional exhaustion	0.237	0.231	0.352	0.079	0.173	4.479	**<0.001**
Block 3	Symptomatology anxiety	0.459	0.454	1.650	0.143	0.482	11.576	**<0.001**
Block 4	Resilience	0.467	0.461	−0.263	0.103	−0.090	−2.561	**0.011**
Block 5	Psychological well-being	0.474	0.467	-0.869	0.331	−0.100	−2.625	**0.009**

Note. *R*^2^ = Coefficient of determination; Δ*R*^2^ = Variation of *R*^2^; *β* = Unstandardized coefficient; *t* = *t*-value; *n* = 526. Significant coefficients are in bold (*p* < 0.05; *p* < 0.01).

**Table 5:** Coefficients of the anxiety symptoms and emotional exhaustion model in PTSD symptoms: the mediating role of resilience and psychological well-being.

			Estimate	S.E.	IC 90%	*p*-Value
Direct Effects						
Symptomatology anxiety	→	Resilience	−0.241	0.046	[−0.316; −0.163]	***
Symptomatology anxiety	→	Psychological well-being	−0.477	0.036	[−0.536; −0.418]	***
Emotional exhaustion	→	Resilience	−0.247	0.045	[−0.321; −0.025]	***
Emotional exhaustion	→	Psychological well-being	−0.100	0.045	[−0.173; −0.025]	0.025 *
Resilience	→	Symptomatology of PTSD	−0.087	0.032	[−0.139; −0.034]	0.006 **
Psychological well-being	→	Symptomatology of PTSD	−0.101	0.036	[−0.156; −0.038]	0.007 **
Symptomatology anxiety	→	Symptomatology of PTSD	0.478	0.042	[0.405; 0.545]	***
Emotional exhaustion	→	Symptomatology of PTSD	0.176	0.041	[0.107; 0.244]	***
Mediational Model						
Symptomatology anxiety	→	Resilience	−0.241	0.046	[−0.316; −0.163]	***
Symptomatology anxiety	→	Psychological well-being	−0.477	0.036	[−0.536; −0.418]	***
Emotional exhaustion	→	Resilience	−0.247	0.045	[−0.321; −0.173]	***
Emotional exhaustion	→	Psychological well-being	−0.100	0.045	[−0.173; 0.025]	0.025 *
Resilience	→	Symptomatology of PTSD	−0.087	0.032	[−0.139; −0.034]	0.006 **
Psychological well-being	→	Symptomatology of PTSD	−0.101	0.036	[−0.156; −0.038]	0.007 **
Indirect Effects						
Symptomatology anxiety	→	Symptomatology PTSD	0.032	0.010	[0.016; 0.052]	***
Emotional exhaustion	→	Symptomatology PTSD	0.069	0.020	[0.038; 0.102]	***

Note. * *p* < 0.05; ** *p* < 0.01; *** *p* < 0.001.

## Data Availability

The analyzed data are available upon reasonable request.

## References

[1] Brakemeier E.L., Wirkner J., Knaevelsrud C., Wurm S., Christiansen H. (2020). The COVID-19 pandemic as a mental health challenge. J. Clin. Psychol. Psychother..

[2] Koushik N.S. (2020). A population mental health perspective on the impact of COVID19. Psychol. Trauma Theory Res. Pract. Policy.

[3] Charles N.E., Strong S.J., Burns L.C., Bullerjahn M.R., Serafine K.M. (2020). Increased mood disorder symptoms, perceived stress, and alcohol use among college students during the COVID-19 pandemic. PsyArXiv.

[4] Akkaya-Kalayci T., Kothgassner O.D., Wenzel T., Goreis A., Chen A., Ceri V., Özlü-Erkilic Z. (2020). The Impact of the COVID-19 Pandemic on Mental Health and Psychological Well-Being of Young People Living in Austria and Turkey: A Multicenter Study. Int. J. Environ. Res. Public Health.

[5] Majumdar P., Biswas A., Sahu S. (2020). COVID-19 pandemic and lockdown: Cause of sleep disruption, depression, somatic pain, and increased screen exposure of office workers and students of India. Chronobiol. Int..

[6] Laranjeira C., Dixe M., Querido A. (2023). Mental health status and coping among Portuguese higher education students in the early phase of the COVID-19 pandemic. Investig. Health Psychol. Educ..

[7] Rettie H., Daniels J. (2021). Coping and tolerance of uncertainty: Predictors and mediators of mental health during the COVID-19 pandemic. Am. Psychol..

[8] Eisenberg D., Hunt J., Speer N. (2013). Mental health in American colleges and universities: Variation across student subgroups and across campuses. J. Nerv. Ment. Dis..

[9] Patsali M.E., Mousa D.V., Papadopoulou E.V.K., Papadopoulou K.K.K., Kaparounaki C.K., Diakogiannis I., Fountoulakis K.N. (2020). University students’ changes in mental health status and determinants of behavior during the COVID-19 lockdown in Greece. Psychiatry Res..

[10] Papalia D., Feldman R.D. (2013). Desenvolvimento Humano.

[11] Tomova L., Wang K.L., Thompson T., Matthews G.A., Takahashi A., Tye K., Saxe R. (2020). Acute social isolation evokes midbrain craving responses similar to hunger. Nat. Neurosci..

[12] Andrews J.L., Ahmed S., Blakemore S.J. (2020). Navigating the social environment in adolescence: The role of social brain development. Biol. Psychiatry.

[13] Nahar Z., Sohan M., Supti K.F., Hossain M.J., Shahriar M., Bhuiyan M.A., Islam M.R. (2022). Prevalence and associated risk factors for mental health problems among female university students during COVID-19 pandemic: A cross-sectional study findings from Dhaka, Bangladesh. Heliyon.

[14] Brand S., Gerber M., Kalak N., Kirov R., Lemola S., Clough P., Puhse U., Hlosboer-Trachsler E. (2014). Adolescents with greater mental toughness show higher sleep efficiency, more deep sleep and fewer awakenings after sleep onset. J. Adolesc. Health.

[15] Ariño D.O., Bardagi M.P. (2018). Relação entre fatores acadêmicos e a saúde mental de estudantes universitários. Psicol. Em Pesqui..

[16] Menegaldi S.C., Hirdes A., Yamaguchi M., Milani R. (2022). Saúde mental e recursos de enfrentamento em estudantes universitários brasileiros em tempos de pandemia. Avaliação Camp..

[17] Wang C., Zhao H. (2020). The Impact of COVID-19 on Anxiety in Chinese University Students. Front. Psychol..

[18] David I., Schatz E., Myroniuk T.W., Teti M. (2022). “COVID Is Another Layer of Problematic Things”: Change, Vulnerability, and COVID-19 among University Students. Int. J. Environ. Res. Public Health.

[19] Maslach C., Schaufeli W.B., Leiter M.P. (2001). Job Burnout. Annu. Rev. Psychol..

[20] Hawley S.R., Thrivikraman J.K., Noveck N., Romain T.S., Ludy M.J., Barnhart L., Chee W.S.S., Cho M.J., Chong M.H.Z., Du C. (2021). Concerns of college students during the COVID-19 pandemic: Thematic perspectives from the United States, Asia, and Europe. J. Appl. Learn. Teach..

[21] Salanova M., Llorens S. (2008). Estado actual y retos futuros en el estudio del burnout. Papeles Del Psicólogo.

[22] Araoz E.G.E., Ramos N.A.G. (2022). Cansaço emocional em estudantes universitários peruanos no contexto da pandemia de COVID-19. Educ. Form..

[23] Ramos F., Mangá D., Morán C., Escala de Cansancio Emocional para Estudantes Universitários (2005). Propriedades Psicométricas e Associação com Medidas de Personalidade e Saúde Psicológica. Comunicação ao 6º Congresso Virtual de Psiquiatria. Interpsiquis. http://www.psiquiatria.com/interpsiquis2005/.

[24] Maslach C. (2009). Comprendiendo el burnout. Cienc. Trab..

[25] Wu Q., Chi P., Zhang Y. (2023). Association Between Pandemic Fatigue and Subjective Well-Being: The Indirect Role of Emotional Distress and Moderating Role of Self-Compassion. Int. J. Public Health.

[26] Labrague L.J., Ballad C.A. (2021). Lockdown fatigue among college students during the COVID-19 pandemic: Predictive role of personal resilience, coping behaviors, and health. Perspect. Psychiatr. Care.

[27] Jagodics B., Szabó É. (2023). Student burnout in higher education: A demand-resource model approach. Trends Psychol..

[28] Khan A.N. (2024). Students are at risk? Elucidating the impact of health risks of COVID-19 on emotional exhaustion and academic performance: Role of mindfulness and online interaction quality. Curr. Psychol..

[29] Gómez J.F.S., Ruiz E.D., Rivera M.d.J.V., Cárdenas M.H., Mora I.R.H. (2021). Síndrome de burnout en estudiantes de educación superior tecnológica del campus Tierra Blanca en tiempo de COVID-19. RIDE Rev. Iberoam. Para La Investig. Y El Desarro. Educ..

[30] Drăghici A. (2013). Anxiety—General Background of Analysing the Pathological Phenomenon and Normal Emotional Mood. Acta Medica Transilv..

[31] Gritsenko V., Skugarevsky O., Konstantinov V., Khamenka N., Marinova T., Reznik A., Isralowitz R. (2021). COVID 19 Fear, Stress, Anxiety, and Substance Use Among Russian and Belarusian University Students. Int. J. Ment. Health Addict..

[32] Maia B.R., Dias P.C. (2020). Ansiedade, depressão e stresse em estudantes universitários: O impacto da COVID-19. Estud. Psicol..

[33] Gonçalves S.P., Vieira dos Santos J., Silva I.S. (2023). Reflexos da COVID-19 na saúde mental de estudantes universitários. Rev. Psicol..

[34] Conceição V., Rothes I., Gusmão R. (2021). The Association Between Changes in the University Educational Setting and Peer Relationships: Effects in Students’ Depressive Symptoms During the COVID-19 Pandemic. Front. Psychiatry.

[35] Fruehwirth J.C., Biswas S., Perreira K.M. (2021). The COVID-19 pandemic and mental health of first-year college students: Examining the effect of COVID-19 stressors using longitudinal data. PLoS ONE.

[36] American Psychiatric Association (2013). DSM-5 Task Force. Diagnostic and Statistical Manual of Mental Disorders: DSM-5™.

[37] World Health Organization (2020). International Statistical Classification of Diseases and Related Health Problems (ICD-11). https://icd.who.int/en.

[38] Ke S., Sun L., Zhou J., Wang Y., Bu T., Chu H., Yang J., Wang W., Wang W., Li J. (2022). Factors Influencing Post-traumatic Stress Symptoms in Chinese Adolescents During the COVID-19 Pandemic. Front. Psychiatry.

[39] Peng P., Hao Y., Liu Y., Chen S., Wang Y., Yang Q., Wang X., Li M., Wang Y., He L. (2023). The prevalence and risk factors of mental problems in medical students during COVID-19 pandemic: A systematic review and meta-analysis. J. Affect. Disord..

[40] Williams S.N., Armitage C.J., Tampe T., Dienes K. (2020). Public perceptions and experiences of social distancing and social isolation during the COVID-19 pandemic: A UK-based focus group study. BMJ Open.

[41] Fegert J.M., Vitiello B., Plener P.L., Clemens V. (2020). Challenges and burden of the Coronavirus 2019 (COVID-19) pandemic for child and adolescent mental health: A narrative review to highlight clinical and research needs in the acute phase and the long return to normality. Child Adolesc. Psychiatry Ment. Health.

[42] Benson O.M., Whitson M.L. (2022). The protective role of sense of community and access to resources on college student stress and COVID-19-related daily life disruptions. J. Community Psychol..

[43] Zhang C., Shi L., Tian T., Zhou Z., Peng X., Shen Y., Li Y., Ou J. (2022). Associations Between Academic Stress and Depressive Symptoms Mediated by Anxiety Symptoms and Hopelessness Among Chinese College Students. Psychol. Res. Behav. Manag..

[44] American Psychological Association (2019). The Road to Resilience. https://www.apa.org/helpcenter/road-resilience.

[45] Seçer İ., Ulaş S. (2020). The Mediator Role of Academic Resilience in the Relationship of Anxiety Sensitivity, Social and Adaptive Functioning, and School Refusal with School Attachment in High School Students. Front. Psychol..

[46] Miroševič Š., Klemenc-Ketiš Z., Selič P. (2019). The 14-item Resilience scale as a potential screening tool for depression/anxiety and quality of life assessment: A systematic review of current research. Fam. Pract..

[47] Urcos W.H.C., Urcos C.N.C., Ruales E.A.B., Urcos J.F.C. (2020). Stress, anguish, anxiety and resilience of university teachers in the face of COVID-19. Utopía Y Prax. Latinoam..

[48] Brooks S., Amlôt R., Rubin G.J., Greenberg N. (2020). Psychological resilience and post-traumatic growth in disaster-exposed organisations: Overview of the literature. BMJ Mil. Health.

[49] Sturman E.D. (2020). Coping with COVID-19: Resilience and Psychological WellBeing in the Midst of a Pandemic. J. Soc. Clin. Psychol..

[50] Thompson G., McBride R.B., Hosford C.C., Halaas G. (2016). Resilience Among Medical Students: The Role of Coping Style and Social Support. Teach. Learn. Med..

[51] Roberts N.J., McAloney-Kocaman K., Lippiett K., Ray E., Welch L., Kelly C. (2021). Levels of resilience, anxiety and depression in nurses working in respiratory clinical areas during the COVID pandemic. Respir. Med..

[52] Serrão C., Castro L., Teixeira A., Rodrigues A.R., Duarte I. (2021). Resilience in Physicians: Contributions to the Validation of the European Portuguese Version of the Resilience Scale. Acta Médica Port..

[53] Duckworth A.L., Peterson C., Matthews M.D., Kelly D.R. (2007). Grit: 46 Perseverance and passion for long-term goals. J. Personal. Soc. Psychol..

[54] Hartley M.T. (2011). Examining the relationships between resilience, mental health, and academic persistence in undergraduate college students. J. Am. Coll. Health.

[55] De Clercq D. (2019). Getting Creative with Resources: How Resilience, Task Interdependence, and emotion sharing mitigate the damage of employee role ambiguity. J. Appl. Behav. Sci..

[56] Yıldırım M., Geçer E., Akgül Ö. (2021). The impacts of vulnerability, perceived risk, and fear on preventive behaviours against COVID-19. Psychol. Health Med..

[57] Xiao W., Liu X., Wang H., Huang Y., Dai Z., Si M., Fu J., Chen X., Jia M., Leng Z. (2023). Mediating role of resilience in the relationship between COVID-19 related stigma and mental health among COVID-19 survivors: A cross-sectional study. Infect. Dis. Poverty.

[58] Hu D., Kong Y., Li W., Han Q., Zhang X., Zhu L.X., Wan S.W., Liu Z., Shen Q., Yang J. (2020). Frontline nurses’ burnout, anxiety, depression, and fear statuses and their associated factors during the COVID-19 outbreak in Wuhan, China: A large-scale cross-sectional study. eClinicalMedicine.

[59] Chi X., Becker B., Yu Q., Willeit P., Jiao C., Huang L., Hossain M.M., Grabovac I., Yeung A., Lin J. (2020). Prevalence and Psychosocial Correlates of Mental Health Outcomes Among Chinese College Students During the Coronavirus Disease (COVID-19) Pandemic. Front. Psychiatry.

[60] Layous K., Chancellor J., Lyubomirsky S. (2014). Positive activities as protective factors against mental health conditions. J. Abnorm. Psychol..

[61] World Health Organization (2001). The World Health Report: 2001: Mental Health: New Understanding, New Hope.

[62] Rustamov E., Nuriyeva U.Z., Allahverdiyeva M., Abbasov T., Mammadzada G., Rustamova N. (2023). Academic self-efficacy, academic procrastination, and well-being: A mediation model with large sample of Azerbaijan. Int. Online J. Prim. Educ..

[63] Huppert F.A. (2009). Psychological well-being: Evidence regarding its causes and consequences. Appl. Psychol. Health Well-Being.

[64] Ruggeri K., Garcia-Garzon E., Maguire Á., Matz S., Huppert F.A. (2020). Well-being is more than happiness and life satisfaction: A multidimensional analysis of 21 countries. Health Qual. Life Outcomes.

[65] de Figueiredo C.S., Sandre P.C., Portugal L.C.L., Mázala-de-Oliveira T., da Silva Chagas L., Raony Í., Ferreira E.S., Giestal-De-Araujo E., dos Santos A.A., Bomfim P.O.-S. (2020). COVID-19 pandemic impact on children and adolescents’ mental health: Biological, environmental, and social factors. Prog. Neuro-Psychopharmacol. Biol. Psychiatry.

[66] Cardenas M.C., Bustos S.S., Chakraborty R. (2020). A ‘parallel pandemic’: The psychosocial burden of COVID-19 in children and adolescents. Acta Pediatr..

[67] Afonso P. (2020). The impact of the COVID-19 pandemic on mental health. Acta Médica Port..

[68] Nishiura H., Jung S.M., Linton N.M., Kinoshita R., Yang Y., Hayashi K., Kobayashi T., Yuan B., Akhmetzhanov A.R. (2020). The Extent of Transmission of Novel Coronavirus in Wuhan, China, 2020. J. Clin. Med..

[69] Karatzias T., Shevlin M., Murphy J., McBride O., Ben-Ezra M., Bentall R.P., Vallières F., Hyland P. (2020). Posttraumatic Stress Symptoms and Associated Comorbidity During the COVID-19 Pandemic in Ireland: A Population-Based Study. J. Trauma. Stress.

[70] Xiong J., Lipsitz O., Nasri F., Lui L.M.W., Gill H., Phan L., Chen-Li D., Iacobucci M., Ho R., Majeed A. (2020). Impact of COVID-19 pandemic on mental health in the general population: A systematic review. J. Affect. Disord..

[71] Dragan M., Grajewski P., Shevlin M. (2021). Adjustment disorder, traumatic stress, depression and anxiety in Poland during an early phase of the COVID-19 pandemic. Eur. J. Psychotraumatology.

[72] Ala S., Ramos-Campos F., Relva I.C. (2024). Emotional Exhaustion Scale (ECE): Psychometric Properties in a Sample of Portuguese University Students. Eur. J. Investig. Health Psychol. Educ..

[73] Hoyt L.T., Cohen A.K., Dull B., Castro E.M., Yazdani N. (2021). “Constant stress has become the new normal”: Stress and anxiety inequalities among US college students in the time of COVID-19. J. Adolesc. Health.

[74] Wenjuan G., Siging P., Xingiao L. (2020). Gender differences in depression, anxiety, and stress among college students: A longitudinal study from China. J. Afettctive Disord..

[75] Di Corrado D., Muzii B., Magnano P., Coco M., La Paglia R., Maldonato N.M. (2022). The Moderated Mediating Effect of Hope, Self-Efficacy and Resilience in the Relationship between Post-Traumatic Growth and Mental Health during the COVID-19 Pandemic. Healthcare.

[76] Liu N., Zhang F., Wei C., Jia Y., Shang Z., Sun L., Wu L., Sun Z., Zhou Y., Wang Y. (2020). Prevalence and predictors of PTSS during COVID-19 outbreak in China hardest-hit areas: Gender differences matter. Psychiatry Res..

[77] Zeng F., John W.C.M., Sun X., Wang Y. (2023). COVID-19-associated impact and post-traumatic stress symptoms 39 days after pandemic in a sample of home-quarantined Chinese college students: The mediating effecting of past stressful events, psychological resilience, and social support. BMC Psychiatry.

[78] Shanahan L., Steinhoff A., Bechtiger L., Murray A.L., Nivette A., Hepp U., Ribeaud D., Eisner M. (2022). Emotional distress in young adults during the COVID-19 pandemic: Evidence of risk and resilience from a longitudinal cohort study. Psychol. Med..

[79] Aguiar F., Barroso I., Monteiro M.J., Pereira M.d.C., Caramelo A. (2024). Psychological well-being in nursing students: Relationship with gender and age. Motricidade.

[80] Jiang Y., Yi Z., Yao Y., Hu Y., Li F., Ma H. (2023). Effects of college students’ mindfulness on depression symptoms during the epidemic prevention and control period: The mediating effect of psychological resilience. Front. Psychiatry.

[81] Holm-Hadulla R.M., Klimov M., Juche T., Möltner A., Herpertz S.C. (2021). Well-Being and Mental Health of Students during the COVID-19 Pandemic. Psychopathology.

[82] Serpa-Barrientos A., Calvet M.L.M., Acosta A.G.D. (2023). The relationship between positive and negative stress and posttraumatic growth in university students: The mediating role of resilience. BMC Psychol..

[83] Soga M., Evans M.J., Tsuchiya K., Fukano Y. (2021). A room with a green view: The importance of nearby nature for mental health during the COVID-19 pandemic. Ecol. Appl..

[84] Pouso S., Borja Á., Fleming L.E., Gómez-Baggethun E., White M.P., Uyarra M.C. (2021). Contact with blue-green spaces during the COVID-19 pandemic lockdown beneficial for mental health. Sci. Total Environ..

[85] Weathers F.W., Litz B.T., Keane T.M., Palmieri P.A., Marx B.P., Schnurr P.P. (2013). The PTSD Checklist for DSM-5 (PCL-5)—Standard [Measurement Instrument]. https://www.ptsd.va.gov/.

[86] Carvalho T., da Motta C., Pinto-Gouveia J. (2020). Portuguese version of the Posttraumatic Stress Disorder Checklist for DSM-5 (PCL-5): Comparison of latent models and other psychometric analyses. J. Clin. Psychol..

[87] Blevins C.A., Weathers F.W., Davis M.T., Witte T.K., Domino J.L. (2015). The Posttraumatic Stress Disorder Checklist for DSM-5 (PCL-5): Development and Initial Psychometric Evaluation. J. Trauma. Stress.

[88] Connor K.M., Davidson J.R.T. (2003). Development of a new resilience scale: The Connor–Davidson Resilience Scale (CD-RISC). Depress. Anxiety.

[89] Almeida M.H., Dias S., Xavier M., Torgal J. (2020). Exploratory and Confirmatory Validation of the Connor-Davidson Resilience Scale (CD-RISC-10) in a Sample of Individuals Registered in Job Centers. Acta Médica Port..

[90] Ribeiro J.L.P. (2011). Inventário de Saúde Mental.

[91] Ribeiro J.L.P. (2001). Inventário de Saúde Mental: Um Estudo de Adaptação para a População Portuguesa. Psicol. Saúde Doenças.

[92] Spitzer R.L., Kroenke K., Williams J.B., Löwe B. (2006). A brief measure for assessing generalized anxiety disorder: The GAD-7. Arch. Intern. Med..

[93] Sousa T.V., Viveiros V., Chai M.V., Vicente F.L., Jesus G., Carnot M.J., Gordo A.C., Ferreira P.L. (2015). Reliability and validity of the Portuguese version of the Generalized Anxiety Disorder (GAD-7) scale. Health Qual. Life Outcomes.

[94] Marôco J. (2018). Análise Estatística com o SPSS Statistics.

[95] Cohen J. (1992). A power primer. Psychol. Bull..

[96] Arbuckle J.L. (2019). Amos, Version 26.0.

[97] Pallant J. (2020). SPSS Survival Manual: A Step by Step Guide to Data Analysis Using IBM SPSS.

[98] Losso A., Ferreira V., Oliveira N., Souza K., Bento P., Silva H. (2022). Sleep and anxiety during the COVID-19 pandemic: A system-1027 atic review. Conjecturas.

[99] Costa C.O., Branco J.C., Vieira I.S., Souza L.D.d.M., da Silva R.A. (2019). Prevalence of anxiety and associated factors in adults. J. Bras. Psiquiatr..

[100] Haesebaert F., Haesebaert J., Zante E., Franck N. (2020). Who maintains good mental health in a locked-down country? A French nationwide online survey of 11,391 participants. Health Place..

[101] World Health Organization (2019). Gender and Health. https://www.who.int/health-topics/gender#tab=tab_1.

[102] Farhane-Medina N.Z., Luque B., Tabernero C., Castillo-Mayén R. (2022). Factors associated with gender and sex differences in anxiety prevalence and comorbidity: A systematic review. Sci. Prog..

[103] Hodes G.E., Epperson C.N. (2019). Sex differences in vulnerability and resilience to stress across the life span. Biol. Psychiatry.

[104] Gitay M.N., Fatima S., Arshad S., Arshad B., Ehtesham A., Baig M.A., Ilyas M.F., Rizvi S.F., Farooqui Q., Masroor M. (2019). Gender Differences and Prevalence of Mental Health Problems in Students of Healthcare Units. Community Ment. Health J..

[105] Cao W., Fang Z., Hou G., Han M., Xu X., Dong J., Zheng J. (2020). The psychological impact of the COVID-19 epidemic on college students in China. Psychiatry Res..

[106] Elharake J.A., Akbar F., Malik A.A., Gilliam W., Omer S.B. (2023). Mental Health Impact of COVID-19 among Children and College Students: A Systematic Review. Child Psychiatry Hum. Dev..

[107] Ribeiro A.I., Triguero-Mas M., Santos C.J., Gómez-Nieto A., Cole H., Anguelovski I., Silva F.M., Baró F. (2021). Exposure to nature and mental health outcomes during COVID-19 lockdown. A comparison between Portugal and Spain. Environ. Int..

[108] Setiawati Y., Wahyuhadi J., Joestandari F., Maramis M.M., Atika A. (2021). Anxiety and resilience of healthcare workers during COVID-19 pandemic in Indonesia. J. Multidiscip. Healthc..

[109] Killgore W.D.S., Taylor E.C., Cloonan S.A., Dailey N.S. (2020). Psychological resilience during the COVID-19 lockdown. Psychiatry Res..

[110] Mosheva M., Hertz-Palmor N., Dorman Ilan S., Matalon N., Pessach I.M., Afek A., Ziv A., Kreiss Y., Gross R., Gothelf D. (2020). Anxiety, pandemic-related stress and resilience among physicians during the COVID-19 pandemic. Depress. Anxiety.

[111] Ran L., Wang W., Ai M., Kong Y., Chen J., Kuang L. (2020). Psychological resilience, depression, anxiety, and somatization symptoms in response to COVID-19: A study of the general population in China at the peak of its epidemic. Soc. Sci. Med..

[112] Barzilay R., Moore T.M., Greenberg D.M., DiDomenico G.E., Brown L.A., White L.K., Gur R.C., Gur R.E. (2020). Resilience, COVID-19-related stress, anxiety and depression during the pandemic in a large population enriched for healthcare providers. Transl. Psychiatry.

[113] Zhang W.R., Wang K., Yin L., Zhao W.F., Xue Q., Peng M., Min B.Q., Tian Q., Leng H.X., Du J.L. (2020). Mental Health and Psychosocial Problems of Medical Health Workers during the COVID-19 Epidemic in China. Psychother. Psychosom..

[114] Freire C., Ferradás M.M., Valle A., Núñez J.C., Vallejo G. (2016). Profiles of Psychological Well-being and Coping Strategies among University Students. Front. Psychol..

[115] Kira I.A., Shuwiekh H.A.M., Ashby J.S., Elwakeel S.A., Alhuwailah A., Sous M.S.F., Baali S.B.A., Azdaou C., Oliemat E.M., Jamil H.J. (2023). The Impact of COVID-19 Traumatic Stressors on Mental Health: Is COVID-19 a New Trauma Type. Int. J. Ment. Health Addict..

[116] Horesh D., Brown A.D. (2020). Traumatic stress in the age of COVID-19: A call to close critical gaps and adapt to new realities. Psychol. Trauma Theory Res. Pract. Policy.

[117] Nações Unidas (2015). Agenda 2030 de Desenvolvimento Sustentável. Centro de Informação Regional das Nações Unidas para a Europa Ocidental. https://www.instituto-camoes.pt/images/ods_2edicao_web_pages.pdf.

[118] Nações Unidas (2018). Objectivos de Desenvolvimento do Milenio Actualidade. Centro de Informação Regional Das Nações Unidas Para a Europa Ocidental. https://unric.org/pt/objetivos-de-desenvolvimento-sustentavel/.

[119] Gaiotto E., Trapé C., Campos C., FujimoriI E., Carrer F., NichiataI L., Cordeiro L., Bortoli M., Yonekura T., Toma T. (2021). Resposta a necessidades em saúde mental de estudantes universitários: Uma revisão rápida. Rev. Saúde Pública.

[120] Watts A., Van Esbroeck R. (2000). New skills for new futures: A comparative review of higher education guidance and counselling services in the European Union. Int. J. Adv. Couns..

[121] Sahão F., Kienen N. (2021). Adaptação e saúde mental do estudante universitário: Revisão sistemática da literatura. Psicol. Esc. Educ..

[122] Savoji A., Ganji K. (2013). Increasing mental health of university students through Life Skills Training (LST). Procedia Soc. Behav. Sci..

[123] Castro V. (2017). Reflexões sobre a saúde mental do estudante universitário: Estudo empírico com estudantes de uma instituição pública de ensino superior. Rev. Gestão Em Foco..

[124] Cortez E., Braga A., Oliveira A., Ribas B., Mattos M., Marinho T., Cavalcanti T., Dutra V. (2017). Promoção à saúde mental dos estudantes universitários. Rev. PróuniverSUS.

[125] Gundim V., Santos F., Santos J., Vasconcellos E., Sousa R. (2021). Saúde mental de estudantes universitários durante a pandemia de COVID-19. Rev. Baiana Enferm..

